# Research Progress on the Regulatory Role of Treg Cells in Inflammatory Eye Diseases

**DOI:** 10.3390/cimb48060555

**Published:** 2026-05-25

**Authors:** Zitong Pan, Yi Wang, Jieya Zhang, Xiaoran Bian, Huaxue Zhang, Jiahao Pan, Xinyu Wang, Dadong Guo

**Affiliations:** 1 College of Ophthalmology and Optometry, Shandong University of Traditional Chinese Medicine, Jinan 250002, China; 17615809693@163.com (Z.P.);; 2 College of Pharmacy, Shandong University of Traditional Chinese Medicine, Jinan 250355, China; 3 Institute for Chinese Medicine and Brain Science, Shandong University of Traditional Chinese Medicine, Jinan 250355, China; 4 Key Laboratory of Traditional Chinese Medicine Classical Theory, Ministry of Education, Jinan 250355, China; 5 Shandong Key Laboratory of Innovation and Application Research in Basic Theory of Traditional Chinese Medicine, Jinan 250355, China; 6 Shandong Provincial Engineering Research Center for the Prevention and Treatment of Major Brain Diseases with Traditional Chinese Medicine, Shandong University of Traditional Chinese Medicine, Jinan 250355, China; 7 Medical College of Optometry and Ophthalmology, Shandong University of Traditional Chinese Medicine, Jinan 250002, China; 8 Shandong Key Laboratory of Integrated Traditional Chinese and Western Medicine for Prevention and Therapy of Ocular Diseases, Jinan 250002, China; 9 Shandong Academy of Eye Disease Prevention and Therapy, Jinan 250002, China

**Keywords:** regulatory T cells, inflammatory eye diseases, immune regulation, signal pathways, targeted intervention

## Abstract

Regulatory T cells (Tregs, CD4^+^ CD25^+^ Foxp3^+^) play a crucial role as a core cell subset in maintaining immune homeostasis in the ocular immune-privileged microenvironment. This review systematically summarizes the stage-specific regulatory mechanisms of Treg cells in common inflammatory diseases such as keratitis, uveitis, and dry eye syndrome, including intercellular interactions, signal pathway mediation, and cytokine network regulation, as well as key experimental evidence (animal/cell models and clinical sample data) and research progress in targeted therapy. Studies have shown that Treg cells maintain ocular immune balance by secreting anti-inflammatory cytokines (such as IL-10 and TGF-β), regulating signaling pathways (STAT, PI3K/AKT, SIRT1, etc.), and interacting with immune cells (macrophages, dendritic cells). Their functions are regulated by multiple factors such as cytokine networks, epigenetic modifications, and delivery vectors. Targeted interventions based on Treg cells (cell therapy, drug intervention, and signaling pathway regulation) and combined treatment strategies have shown good anti-inflammatory potential. This article, in light of current research limitations (such as insufficient analysis of cell heterogeneity and the disconnect between basic and clinical research), proposes future research directions, providing a theoretical basis for the understanding of the pathogenesis of inflammatory eye diseases and the development of new immunomodulatory therapies, and establishing a complete research framework of “mechanism–evidence–treatment”.

## 1. Introduction

Inflammatory eye diseases refer to a group of disorders with inflammatory infiltration and immune imbalance in ocular tissues as the core pathological characteristics, including infectious and non-infectious keratitis, uveitis, dry eye syndrome, diabetic retinopathy, and inflammation related to retinal detachment [1,2]. The global incidence of these diseases remains high, and their core pathological changes such as corneal stromal necrosis, disruption of the blood–aqueous barrier, and damage to retinal nerve cells often lead to irreversible visual impairment and even blindness in severe cases [1,3]. Traditional clinical treatments mainly rely on broad-spectrum anti-inflammatory drugs (e.g., glucocorticoids) and immunosuppressants, but long-term use of these drugs can cause serious complications such as systemic immune suppression, increased intraocular pressure, and corneal epithelial damage, and the therapeutic effect is limited for refractory inflammatory eye diseases [2,4]. Therefore, it is an urgent clinical need to explore the core regulatory mechanism of ocular inflammatory immune imbalance and develop precise and low-toxicity targeted immunomodulatory therapies. The eye possesses unique immune privilege characteristics which are fundamental to this pursuit. This privilege, characterized by low immunogenicity, enrichment of anti-inflammatory factors, and physical isolation by the blood–eye barrier, creates a “low-inflammatory interference” microenvironment. This microenvironment is not merely a passive feature but an active prerequisite that enables key immunoregulatory cells, such as regulatory T cells (Tregs), to function effectively without being overwhelmed by pro-inflammatory signals, thereby maintaining ocular immune homeostasis.

The ocular immune privilege is a complex system maintained by multiple, equally important components that work in concert. Tregs are a key subset of cells responsible for maintaining immune tolerance in the immune system, a crucial executor within this system, specifically inhibiting the activation and proliferation of effector T cells (Th1, Th17, CD8^+^ T cells) through direct cell contact inhibition and the secretion of anti-inflammatory cytokines such as interleukin-10 (IL-10) and transforming growth factor-beta (TGF-β), thereby preventing pathological damage to tissues caused by excessive immune responses [5,6]. The eye has unique immune privilege characteristics, with a core of low immunogenicity, enrichment of anti-inflammatory cytokines, and physical isolation by the blood–eye barrier, providing a “low inflammatory interference” microenvironmental basis for Treg cells to function [1,3,7]. Factors such as TGF-β and retinoic acid (RA) in the aqueous humor can induce the differentiation of naive T cells into Tregs [3] and resident cells in the eye (corneal endothelial cells, retinal pigment epithelial cells) further enhance the immunosuppressive microenvironment by expressing molecules such as programmed death-ligand 1 (PD-L1) and cytotoxic t-lymphocyte-associated protein 2 alpha (CTLA-2α) [8,9]. These factors are equally important as Tregs; they form an interdependent network where, for instance, aqueous humor factors induce Treg differentiation, and Tregs, in turn, help maintain the anti-inflammatory milieu.

In recent years, numerous studies have confirmed that Treg cells play a dual role in the pathological process of inflammatory eye diseases—maintaining ocular immune homeostasis under physiological conditions, while a reduction in their number or functional defects in pathological conditions can exacerbate the progression of inflammation [1,10,11]; enhancing the function of Treg cells through targeted intervention can effectively alleviate ocular inflammation [7,12,13]. Our research team holds that Treg cells are the core regulatory hub of ocular immune homeostasis, and the imbalance of Treg/effector T cell is a common pathological mechanism of various inflammatory eye diseases, which makes Treg cells an ideal target for the treatment of inflammatory eye diseases. However, the regulatory mechanisms of Treg cells in different inflammatory eye diseases show significant heterogeneity (e.g., in corneal transplant rejection, Tregs primarily function to inhibit effector T cells and angiogenesis to maintain graft tolerance. In contrast, in herpes simplex keratitis, a subset of Tregs converts into pro-inflammatory ex-Tregs, exacerbating disease. Similarly, in autoimmune uveitis, enhancing Treg function via IL-2 is therapeutic, whereas in dry eye disease, Treg dysfunction is closely linked to dysregulated Substance P and IL-6 signaling) and the key factors influencing their function and targeted treatment strategies still need to be systematically integrated. Based on this, this review adopts a “mechanism–evidence–treatment” logical framework to systematically summarize the physiological distribution and immune homeostasis maintenance mechanisms of Treg cells in the eye, their regulatory roles and experimental evidence in different inflammatory eye diseases, the factors influencing their function, and also to summarize the targeted treatment strategies based on Treg cells. At the same time, it analyzes the current research limitations and proposes future directions, providing a comprehensive reference for the basic research and clinical translation of inflammatory eye diseases.

## 2. The Physiological Distribution of Treg Cells in the Eye and the Mechanism of Maintaining Immune Homeostasis

### 2.1. Immunologic Characteristics of Ocular Microenvironment

The eye has typical immune privilege features, with the core characteristics being low immunogenicity, enrichment of anti-inflammatory cytokines, and the physical isolation effect of the blood–eye barrier (such as the blood–retinal barrier and the blood–corneal barrier), providing a low-inflammatory interference microenvironment for Treg cells to exert their immune regulatory functions [1,2,7,14,15]. Aqueous humor is rich in immune regulatory factors such as TGF-β, retinoic acid (RA), and α-melanocyte-stimulating hormone (α-MSH), which can induce the differentiation of naive T cells into Treg cells and inhibit the secretion of pro-inflammatory factors by effector T cells [3,9]; ocular resident cells (such as corneal endothelial cells, retinal pigment epithelial cells, and glial Müller cells) participate in the construction of an immunosuppressive microenvironment by expressing molecules such as PD-L1, CTLA-2α, and glucocorticoid-induced TNF receptor ligand (GITRL), or by secreting immune regulatory factors [3,9].

Under pathological conditions, the ocular microenvironment undergoes dynamic changes: in conditions such as corneal transplantation trauma and immune rejection, myopia-related corneal tissue inflammation, and diabetic retinopathy, the integrity of the blood–eye barrier is disrupted, with increased infiltration of inflammatory cells and the release of pro-inflammatory factors (such as interleukin-6 (IL-6), tumor necrosis factor-alpha (TNF-α), interleukin-17 (IL-17), etc.), thereby disrupting local immune homeostasis [1,16,17]; anterior chamber-associated immune deviation (ACAID), as a key regulatory process of ocular immune privilege, can induce the generation of Treg cells after antigen-presenting cells (APCs) in the iris and ciliary body capture antigens, maintaining immune homeostasis such as corneal transplant tolerance [18]; when lipopolysaccharide (LPS)-induced inflammation occurs, the integrity of the blood–retinal barrier is compromised, and a large amount of pro-inflammatory cytokines are released, further exacerbating immune imbalance [19]; in the lacrimal glands of aged mice, there is lymphocyte infiltration and the formation of tertiary lymphoid-like tissue, and the interaction between inflammatory factors and immune cells affects the phenotype and function of Treg cells [20]; and in the microenvironment of conjunctival squamous cell carcinoma, the immune response between tumor cells and stromal cells remodels the local inflammatory microenvironment and indirectly regulates the infiltration and activation of Treg cells [15].

### 2.2. The Specific Distribution of Treg Cells in Ocular Tissues

Treg cells are specifically distributed in various ocular tissues and related lymphoid organs, and their distribution characteristics change dynamically under inflammatory conditions. Understanding their distribution patterns under physiological homeostasis—as well as the dynamic changes in their distribution under pathological conditions such as inflammation, tumors, and immune senescence, and marking the detection techniques and associated diseases for each tissue distribution study—will provide fundamental data for histological research on the role of Treg cells in ocular immune regulation. The specific summary is presented in Table 1.

### 2.3. The Molecular Mechanism of Treg Cells in Maintaining Ocular Immune Homeostasis

**Figure 1:**
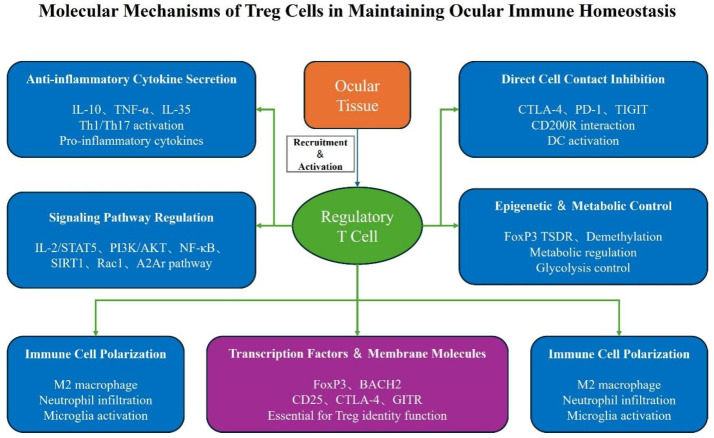
Molecular mechanism of Treg cells in maintaining ocular immune homeostasis.

Treg cells maintain ocular immune homeostasis through a complex and multi-dimensional regulatory network. Our research team summarizes that this multi-dimensional regulatory network has the characteristics of “interconnection and mutual regulation”, and each regulatory mechanism is not independent but interacts with each other to form a complete regulatory system, which ensures the stable play of the immunosuppressive function of Treg cells in the ocular immune-privileged microenvironment. Figure 1 shows a schematic illustration of the molecular mechanisms by which Treg cells maintain ocular immune homeostasis.

#### 2.3.1. Anti-Inflammatory Cytokine Secretion

Secreting anti-inflammatory factors such as IL-10, TGF-β and interleukin-35 (IL-35) is the most basic and important regulatory mechanism of Treg cells. These cytokines can directly inhibit the activation and proliferation of effector T cells (Th1, Th17, etc.) and reduce the release of pro-inflammatory factors such as interferon-gamma (IFN-γ), IL-17 and TNF-α, thereby directly inhibiting the ocular inflammatory response. In the EAU model, IL-10 can specifically suppress the inflammatory response mediated by Th17 cells, and TGF-β can induce ocular immune tolerance by promoting the differentiation of naive T cells into Treg cells [2,14,31,48,49].

#### 2.3.2. Direct Contact Inhibition of Cells

Treg cells realize the immunosuppressive effect through direct interaction with surface molecules (CTLA-4, PD-1, T- cell immunoreceptor with ig and itim domains (TIGIT), cluster of differentiation 200 receptor (CD200R), etc.) and effector T - cells, dendritic cells (DCs) and other immune cells: on the one hand, they directly inhibit the activation of effector T - cells through surface molecule interaction; on the other hand, they downregulate the expression of CD80/86 on the surface of DCs, induce DC apoptosis, and inhibit the initiation of immune responses from the source. After corneal transplantation, Treg cells interact with CD200R+ regulatory DCs to promote the formation of immune tolerance and reduce the risk of graft rejection [14,17,25,33,48].

#### 2.3.3. Signal Pathway Regulation

A variety of signal pathways form a complex regulatory network to participate in the regulation of the survival, differentiation and function of Treg cells. The IL-2 signaling pathway maintains the survival and homeostasis of Treg cells, and CsA can enhance this pathway to reverse the inflammatory phenotype of Treg cells [2]; the phosphatidylinositol 3-kinase/protein kinase b (PI3K/AKT), signal transducer and activator of transcription 3/5 (STAT 3/5), nuclear factor kappa b (NF-κB), sirtuin 1 (SIRT1), proviral integration site for moloney murine leukemia virus 1/protein kinase b/forkhead box o1 (PIM1/AKT/FOXO1), and other pathways are involved in regulating the differentiation and function of Treg cells; NAD+ regulates the balance of Th1/Th17/Tregs by activating the SIRT1 signaling pathway [13,14,33,39,49]; the Rac1 signaling pathway regulates the recruitment and stability of Treg cells [21,35,48]; the A2Ar signaling pathway regulates the homing and function of Treg subsets [50]. Our research team found that the cross-regulation of these signal pathways is the key to maintaining the stable function of Treg cells, and the abnormal activation or inhibition of a single pathway will lead to the functional impairment of Treg cells.

#### 2.3.4. Epigenetics and Metabolic Regulation

As a core transcription factor, the expression stability of forkhead box p3 (FOXP3) depends on the demethylation and acetylation modifications of the treg-specific demethylated region (TSDR) (such as SAHA promoting FOXP3 acetylation) [24,31,51]; Treg cells maintain their immunosuppressive phenotype and regulate the functions of immune cells by modulating glycolysis, alanine metabolism, arginine–proline metabolism, etc. [52,53].

#### 2.3.5. Immune Cell Polarization Regulation

Treg cells promote the polarization of macrophages to the M2 anti-inflammatory phenotype through direct contact and paracrine signaling [11,34,54]. They can secrete TGF-β and IL-10, which bind to corresponding receptors on macrophages, driving the upregulation of M2 markers like CD206 and Arg1 [11,34,54]. For example, in a model of autoimmune dacryoadenitis, treatment with MSC-derived extracellular vesicles enhanced Treg function, which subsequently shifted the macrophage population from a pro-inflammatory M1-dominant to a reparative M2-dominant state, alleviating glandular inflammation [6]. Similarly, in wet age-related macular degeneration, melatonin-induced recruitment of Tregs was associated with an increased proportion of M2 macrophages [5,11,34].

#### 2.3.6. Cytokine Networks and Chemokine Receptor-Mediated

Homeostasis is achieved by homing to ocular tissues and lymphoid organs through chemokine receptors such as CXCR3, CCR4, CCR6/CCR7; regulation of the CXCL9/CXCR3 signaling axis inhibits excessive reactivity of astrocytes [34,50].

#### 2.3.7. Transcription Factors and Membrane Molecules Regulation

Transcription factors such as Foxp3 and btb and cnc homology 2 (BACH2) enhance the immunosuppressive activity of Treg cells [55]; membrane molecules such as CD25, CTLA-4, and GITR are involved in maintaining the function of Treg cells, and the deficiency of CD25 leads to a reduction in the number and functional defects of Treg cells [5,6].

## 3. The Regulatory Role of Treg Cells in Different Inflammatory Eye Diseases and Experimental Evidence

Treg cells play an important regulatory role in the occurrence and development of various inflammatory eye diseases, and the core pathological feature of various inflammatory eye diseases is the imbalance of Treg/effector T cell ratio and the functional impairment of Treg cells. However, due to the different pathological characteristics of and microenvironmental changes in different ocular tissues, the regulatory mechanisms of Treg cells in different inflammatory eye diseases show significant tissue specificity and disease specificity. Our research team summarizes that the regulatory role of Treg cells in inflammatory eye diseases is mainly reflected in three aspects: on the one hand, in the early stage of inflammation, the number of Treg cells increases in a compensatory manner to inhibit excessive inflammatory response and prevent the spread of inflammation; on the other hand, in the middle and late stages of persistent inflammation, the pro-inflammatory microenvironment leads to the functional impairment or quantitative reduction in Treg cells, which further exacerbates the inflammatory response and leads to the chronicity and progression of the disease. Furthermore, a third, critical aspect is the functional plasticity and pro-inflammatory conversion of Tregs. Under specific inflammatory conditions (e.g., viral infection), a subset of Tregs can lose their suppressive function and transform into pro-inflammatory “ex-Treg” cells, directly exacerbating tissue damage. This conversion, along with other phenotypic shifts such as the acquisition of an effector-memory phenotype (CD44hi) in chronic inflammation, represents a distinct “functional reshaping” of Tregs beyond mere compensation or exhaustion. In this section, we systematically summarize the regulatory mechanisms and key experimental evidence of Treg cells in common inflammatory eye diseases such as corneal inflammatory diseases, uveitis, conjunctivitis, retinal choroiditis and other inflammatory eye diseases, and clarify the disease-specific regulatory characteristics of Treg cells (Table 1).

### 3.1. Corneal Inflammatory Diseases

The cornea is the outermost tissue of the eye and is the first to be exposed to external stimuli and pathogens, so corneal inflammatory diseases are the most common inflammatory eye diseases. Treg cells play a key regulatory role in the occurrence and development of corneal inflammatory diseases, and the functional impairment of Treg cells is the core link leading to the persistence and progression of corneal inflammation.

#### 3.1.1. Corneal Transplant Rejection Regulatory Mechanism

Corneal transplant rejection is mainly mediated by CD4^+^ T cells, and the balance between Th17 and Treg cells is a key factor determining the survival of the graft [12,17]; Treg cells reduce the risk of graft rejection by inhibiting corneal angiogenesis, lymph angiogenesis, and immune cell infiltration; they secrete anti-inflammatory factors such as IL-10 to protect corneal endothelial cells (CEnCs) and inhibit CEnC apoptosis induced by IFN-γ and TNF-α [3,7,12,17,47,56]; the anterior chamber can induce the generation of allograft antigen-specific Treg cells. By suppressing local allogeneic immune responses and reducing the levels of inflammatory factors, the survival of the graft can be prolonged [3]. A variety of animal experiments have confirmed the significant anti-rejection effect of enhancing Treg cell function in corneal transplantation, which provides a reliable experimental basis for the clinical application of Treg cell-based therapy for corneal transplant rejection [3,12,17,47].

#### 3.1.2. Herpes Simplex Keratitis (HSK)

After HSV-1 infection, the CD25lo Treg subset in the cornea is prone to transforming into ex-Treg, secreting IFN-γ and promoting the progression of corneal stromal inflammation; IL-12 is a key pro-inflammatory factor driving this transformation via the IL-12/STAT4/T-bet axis. Specifically, high levels of IL-12 in the corneal microenvironment bind to the IL-12 receptor on CD25lo Tregs, leading to phosphorylation of STAT4. Activated STAT4 upregulates the expression of the transcription factor T-bet, which suppresses Foxp3 expression. This transcriptional reprogramming causes the loss of the suppressive phenotype and drives the conversion into IFN-γ-secreting ex-Tregs; corneal-resident pDCs maintain the stability of Treg through the TLR9-IFN-α axis [23,24].

#### 3.1.3. Other Corneal Inflammations

In Aspergillus fumigatus keratitis, CD3ε enhances the anti-inflammatory effect of Treg by upregulating the expression of IL-10, thereby inhibiting the progression of corneal ulcers; the absence of CD3ε leads to damage in Treg-related anti-inflammatory pathways and aggravates corneal tissue damage [57]. In Fuchs’ endothelial corneal dystrophy (FECD), transient receptor potential vanilloid 1 (TRPV1) is negatively correlated with Treg cells and affects Treg function by regulating calcium transport and inflammatory pathways; the TRPV1 antagonist caps azepine can upregulate the expression of Treg markers and inhibit inflammation [21]. After corneal nerve ablation, substance P is released, downregulating the expression of CD103 and IFN-γ receptor on Treg cells and inhibiting their function; neurokinin 1 receptor (NK-1R) antagonists can block this effect [9].

### 3.2. Uveitis

Despite diverse etiologies (e.g., autoimmune, infectious), a common core pathological mechanism underpinning various forms of uveitis is the imbalance between pro-inflammatory effector T cells (particularly Th17) and regulatory T (Treg) cells. Uveitis is a group of severe intraocular inflammatory diseases that can involve multiple ocular tissues such as the iris, ciliary body and choroid, and is one of the main causes of severe visual impairment in young and middle-aged people. The core pathological mechanism of uveitis is the imbalance of Th17/Treg cell equilibrium, and the functional impairment of Treg cells is the key factor leading to the occurrence and development of uveitis [33,39,50,55].

#### 3.2.1. Experimental Autoimmune Uveitis (EAU)

The core pathological mechanism of EAU is the imbalance of Th17/Treg cell equilibrium; Treg cells inhibit the activation of Th17 cells and secrete IL-10 and other anti-inflammatory factors to reduce retinal inflammation; A2Ar-dependent Treg subsets (PD-1+, TIGIT+) exert their functions by homing to the eye and lymphoid tissues through CCR6/CCR7; the PIM1/AKT/FOXO1 pathway inhibits Treg cell function, and inhibiting PIM1 can restore their activity [33,39,50,55]; progesterone can enhance the stability and immunosuppressive activity of Treg cells by upregulating the expression of functional molecules such as TGFBR2, BACH2, and IL-10 in Treg cells, while simultaneously inhibiting the activation of the Id2/Pim1 axis in Th17 cells [55]. A variety of targeted intervention strategies to enhance Treg cell function have been proved to effectively alleviate EAU and improve retinal function, which provides a good experimental basis for the treatment of clinical autoimmune uveitis [30,33,50,55].

#### 3.2.2. Tuberculous Uveitis

The frequency of Treg cells in the peripheral blood of patients with tuberculous uveitis was significantly reduced, with downregulated expression of TGF-β and IL-2Rα (CD25), and weakened inhibitory function on Th1 and Th17 cells; the frequency of peripheral Treg cells was negatively correlated with the levels of IFN-γ and IL-17A in the eye [37]. Clinical sample detection shows that the levels of pro-inflammatory factors such as IFN-γ and IL-17A in the vitreous fluid of patients with tuberculous uveitis are significantly elevated, which is the direct cause of intraocular tissue damage [37].

#### 3.2.3. Chronic Autoimmune Uveitis (CAU)

The dominance of memory Th17 cells and the weakened inhibitory effect of Treg cells lead to chronic inflammation [4]. In the CAU model, the proportion of CD44 hiIL-17^+^ T cells in the retina and lymphoid tissues increases, and the function of Tregs is relatively defective [4].

### 3.3. Conjunctivitis

A common thread in conjunctivitis, regardless of cause, involves the dysfunction or altered function of Treg cells. In allergic conjunctivitis, this manifests as a quantitative reduction and functional impairment of Tregs. In contrast, in conjunctival squamous cell carcinoma-related inflammation, Tregs are recruited and their function is co-opted within the tumor microenvironment to suppress anti-tumor immunity. Thus, the “dysregulation of Treg function” is a shared feature.

#### 3.3.1. Allergic Conjunctivitis (AC)

The frequency of Treg cells in the peripheral blood of patients with allergic conjunctivitis was significantly reduced, and the expression of SLAM molecules on the surface of CD4^+^ T cells was upregulated, disrupting the balance between Treg and effector T cells; miR-146a enhanced the inhibitory effect of Treg on effector T cells by inhibiting the NF-κB signaling pathway; α-MSH could induce the differentiation of Treg cells and downregulate the levels of pro-inflammatory factors [22,43,58]. Clinical and animal experiments have confirmed that enhancing the function of Treg cells can effectively reduce the symptoms of allergic conjunctivitis and inhibit the ocular surface allergic inflammatory response [43,44,58].

#### 3.3.2. Conjunctival Squamous Cell Carcinoma-Related Conjunctivitis

The Foxp3/CXCR4 axis mediates the infiltration and activation of Treg cells, inhibits anti-tumor immunity, and promotes tumor progression [15]. In 31 cases of human conjunctival squamous cell carcinoma tissues, the proportion of Foxp3/CXCR4 double-positive Treg cells in the Tadv group was increased and was associated with progression-free survival (*p* = 0.049) [15].

### 3.4. Retinal Choroiditis

#### 3.4.1. Wet Age-Related Macular Degeneration (AMD)

In patients with wet AMD, the Rac1 signaling pathway in Treg cells is abnormally activated, promoting the secretion of IL-10 and TGF-β1 and mediating the formation of choroidal neovascularization; melatonin promotes CCR4-mediated Treg recruitment through the tet methylcytosine dioxygenase 2/5′-nucleotidase ecto (TET2/NT5E) axis and regulates the polarization of M1/M2 macrophages [35,36]. Clinical and animal experiments have confirmed that the proportion of Treg cells and the expression of Rac1 in the peripheral blood of patients with wet AMD were significantly higher than those in healthy controls (*p* < 0.0001) [36]; after treatment with melatonin (10 mg/kg) in AMD mice induced by NaIO3, the number of Treg cells in the retina increased by six times and the expression of M2 markers (CD206, Arg1) was elevated [35].

#### 3.4.2. Retinal Detachment

After retinal detachment, the balance of Th17/Treg cells is disrupted, with a decrease in the number and function of Treg cells. Silk fibroin (SF) nanoparticles loaded with dexamethasone (DEX) (SF@DEX) can increase the proportion and function of Treg cells and inhibit the activation of Th17 cells [42]. In the SD rat retinal detachment model, after SF@DEX intervention, the proportion of Treg cells, the expression level of Foxp3, and the levels of IL-10 and TGF-β1 were significantly increased (*p* < 0.001), and the apoptosis rate of retinal ganglion cells was decreased [59].

#### 3.4.3. Oxygen-Induced Retinopathy (OIR)

Hyperoxia leads to retinal ischemia and hypoxia, resulting in a decrease in the number of Tregs and a weakening of their inhibitory function. Low-dose IL-2 can restore the number and function of Tregs and increase the Treg/CD8^+^ T cell ratio. Tregs inhibit microglial activation through CTLA-4-mediated intercellular contact, reducing vascular occlusion and neovascularization [1,11]. In OIR mice, the number of Treg cells increased from 4.41 ± 1.48/field to 10.05 ± 2.91/field (*p* < 0.001), and neovascularization decreased 0.68 times (*p* < 0.01) after low-dose IL-2 treatment [1].

#### 3.4.4. Retinal I/R Injury

Induced mesenchymal stem cells (imsc) promotes the differentiation of Treg cells and inhibits the activation of effector T cells through mitochondrial transport, thereby maintaining retinal immune homeostasis [10]. Intravitreal injection of iMSC (1000 cells/2 μL of normal saline) can significantly increase the number of Foxp3+ Treg cells in the retina, reduce the expression of IL1β, VCAM1, LAMA5, and CCL2 genes, and improve the b-wave amplitude [10].

### 3.5. Other Inflammatory Eye Diseases

#### 3.5.1. Dry Eye Disease (DED)

Dry stress induces an increase in substance P (SP) levels in the draining lymph nodes, which downregulates the expression of Foxp3 and CTLA-4 by binding to NK-1R on the surface of Treg cells, leading to impaired Treg function; IL-6 induces Treg cell dysfunction, and blocking the IL-6 signal can restore its inhibitory effect [25,60]; MDSCs enhance the functional stability of Treg cells by secreting IL-10 [42]. The SP mRNA and protein levels in DLNs of DED mice increased by four times and 25%, respectively. The NK-1R antagonist Spantide I restored Treg inhibitory function and improved corneal fluorescein staining score [25]. After anti-IL-6 antibody treatment, the expression of Foxp3 and CD25 in Treg cells was restored, and the CFS score was significantly decreased (*p* < 0.0001) [60].

#### 3.5.2. Autoimmune Dacryoadenitis

The proportion of Treg cells decreases, the expression of Foxp3 and Nurr1 is downregulated, and the immunosuppressive function is weakened; the imbalance of Th17/Treg contributes to disease progression, and pro-inflammatory factors TNF-α and IL-1β inhibit the function of Treg cells [6]. In the rabbit model of autoimmune dacryoadenitis, compared with the normal group, the proportion of CD4^+^ Foxp3^+^ Treg in the lacrimal gland tissue of the model group was significantly reduced and the expression of Foxp3 mRNA was downregulated by more than 50%. After intervention with hUC-MSC-sEVs, the proportion of Treg increased to 6.94% (3.73% in the control group). The levels of inflammatory factors decreased [6].

#### 3.5.3. Uveal Melanoma-Associated Ocular Inflammation

In the tumor-associated inflammatory microenvironment, IL-6 and IP-10 regulate the infiltration of Treg cells and are involved in the pathological process of tumor-related ocular inflammation [38]. The concentrations of IL-6 and IP-10 in the vitreous fluid of 33 patients with uveal melanoma were significantly elevated and positively correlated with the infiltration of Foxp3+Treg in tumor tissues (*p* = 0.02, 0.03) [38].

## 4. Key Factors Influencing the Function of Treg Cells in Inflammatory Eye Diseases

**Figure 2:**
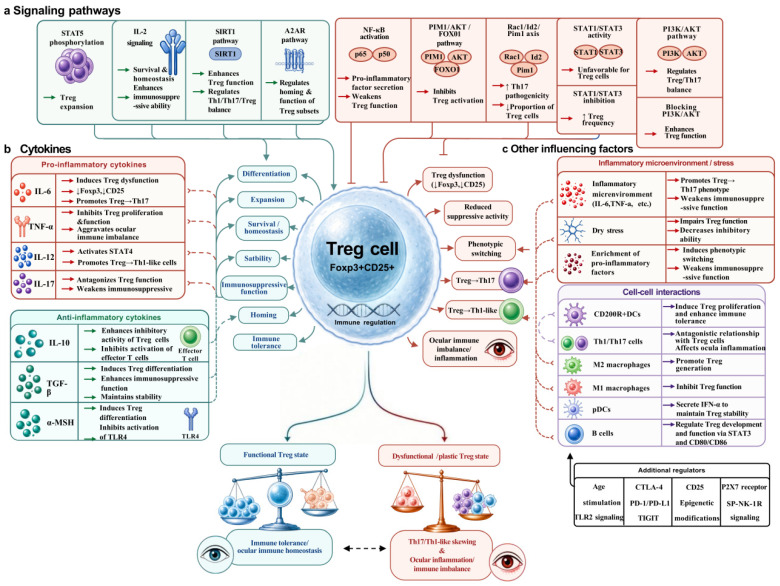
Molecular network mechanism diagram of regulatory T cell (Treg) functional homeostasis and plasticity regulation in the eye.

The factors influencing the function of Treg cells in inflammatory eye diseases are complex and diverse. This study categorizes these factors into drug intervention, signaling pathways, cytokines, microbiota, etc., to clarify the specific mechanisms by which each factor regulates the function of Treg cells and their associated eye diseases, providing a basis for screening targeted intervention points for ocular inflammation that target Treg cells (Table 2 and Figure 2). Our research team summarizes that the functional regulation of Treg cells is the result of the joint action of internal and external factors, and the imbalance of any link will lead to the functional impairment of Treg cells and further induce ocular immune imbalance.

### 4.1. Drug-Related Factors Influencing Treg Function

Pharmacological interventions are the most common and important external factors in regulating the function of Treg cells; a variety of drugs can regulate the number and function of Treg cells by targeting signal pathways, epigenetic modifications and metabolic pathways of Treg cells. Cyclosporin A (CsA) can enhance the IL-2 signaling pathway, reverse the inflammatory phenotype of Treg cells and promote the proliferation of Treg cells [2]; VEGFR1R2Trap can increase the frequency of Treg cells in draining lymph nodes and inhibit the activation of DCs [17]; Apumilast and AS101 can block the PI3K/AKT pathway and inhibit the phosphorylation of AKT and STAT3/4, respectively, thereby promoting the generation and functional enhancement of Treg cells [14,61]; low-dose IL-2 can bind to the IL-2 receptor, promote the proliferation of Treg cells and upregulate the expression of inhibitory molecules (CTLA-4, PD-1, TIGIT) [1,64]; progesterone can upregulate the expression of Treg functional molecules and inhibit the Id2/Pim1 axis, thereby enhancing the stability and immunosuppressive activity of Treg cells [55]; melatonin can activate the TET2/NT5E axis to promote CCR4-mediated Treg recruitment [35].

### 4.2. Signaling Pathways

Signal pathways are the core internal regulatory factors of Treg cell function, and a variety of signal pathways form a complex cross-regulatory network to participate in the regulation of the differentiation, survival and functional play of Treg cells. The PI3K/AKT pathway regulates the balance of Treg/Th17 cells, and blocking this pathway can enhance the function of Treg cells [14,65]; the STAT pathway is an important regulatory pathway of Treg cells—STAT5 phosphorylation induces Treg cell expansion, and STAT1/3 inhibition increases the frequency of Treg cells [14,49]; the IL-2 signaling pathway is the core pathway to maintain the survival and homeostasis of Treg cells and enhance their immunosuppressive ability [2]; the NF-κB pathway can affect the immunosuppressive activity of Treg cells and its activation promotes the secretion of pro-inflammatory factors and weakens the function of Treg cells [65]; the SIRT1 pathway can enhance the function of Treg cells and regulate the balance of Th1/Th17/Tregs after activation [13]; the A2Ar pathway regulates the homing and function of Treg subsets [50]; the PIM1/AKT/FOXO1 pathway inhibits the activation of Treg cells [33]; and the Rac1/Id2/Pim1 axis can improve the pathogenicity of Th17 cells and reduce the proportion of Treg cells [63].

### 4.3. Cytokines

Cytokines are important external regulatory factors that regulate the function of Treg cells, and different cytokines have different regulatory effects on Treg cells, which can be divided into pro-inflammatory cytokines that inhibit Treg cell function and anti-inflammatory cytokines that enhance Treg cell function. IL-6 is a typical pro-inflammatory cytokine that induces Treg cell dysfunction, downregulates the expression of Foxp3 and CD25, and promotes the transformation of Treg cells to Th17 cells [14,16,37,43,60]; TNF-α inhibits the proliferation and function of Treg cells and aggravates ocular immune imbalance [6,14,16,65,66]; IL-12 activates the STAT4 pathway to promote the transformation of Treg cells into Th1-like cells [24]; and IL-17 antagonizes the function of Treg cells and weakens their immunosuppressive effect [2,33]. On the other hand, IL-10 enhances the inhibitory activity of Treg cells and inhibits the activation of effector T cells [2,14,22,67,68,69]; TGF-β induces the differentiation of Treg cells, enhances their immunosuppressive function and maintains their stability [3,8,14,31]; and α-MSH induces the differentiation of Treg cells and inhibits the activation of TLR4 [43].

### 4.4. Other Influencing Factors

The inflammatory microenvironment is an important external factor affecting the function of Treg cells, IL-6, TNF-α and other pro-inflammatory factors in the inflammatory microenvironment promoting the transformation of Treg cells to Th17 phenotype and weakening their immunosuppressive function [14,16]; dry stress leads to impaired Treg cell function and decreased inhibitory ability [25,60]; the enrichment of pro-inflammatory factors induces the phenotypic switching of Treg cells and the loss of immunosuppressive function [18,24]. Cell–cell interaction is also an important factor regulating the function of Treg cells—CD200R+ DCs can induce the proliferation of Treg cells and enhance immune tolerance [7,14,17]; Th1/Th17 cells and Treg cells are antagonistic to each other, which affects the outcome of ocular inflammation [2,14]; M2 macrophages promote the generation of Treg cells, while M1 macrophages inhibit the function of Treg cells [6,11,54]; pDCs secrete IFN-α to maintain the stability of Treg cells [23]; B cells regulate the development and function of Treg cells through STAT3 and CD80/CD86 [68]. In addition, age, antigen-specific stimulation, TLR2 signaling pathway, CTLA-4, PD-1/PD-L1, TIGIT, CD25 molecules, epigenetic modifications, P2X7 receptor and SP-NK-1R signal are also important factors affecting the function of Treg cells [5,9,13,24,25,31,39,50,69,73].

## 5. Treg Cell-Based Therapeutic Strategies for Inflammatory Eye Diseases

Based on the important regulatory role of Treg cells in inflammatory eye diseases, Treg cell-based targeted therapeutic strategies have become a research hotspot in the field of ocular immunology in recent years. These strategies take Treg cells as the core target and realize the treatment of inflammatory eye diseases by enhancing the function of Treg cells, regulating Treg-related signal pathways and optimizing the combination of therapies and targeted delivery systems. Our research team summarizes that Treg cell-based therapeutic strategies have the advantages of high precision, strong specificity and low side effects, and have shown good anti-inflammatory potential in a variety of animal models of inflammatory eye diseases. However, the current therapeutic strategies still face some technical bottlenecks such as low in vitro expansion efficiency of Treg cells, poor ocular targeting of drugs, and disconnection between basic research and clinical translation. In this section, we systematically summarize the latest research progress of Treg cell-based therapeutic strategies for inflammatory eye diseases, including targeted interventions to enhance Treg cell function (cell therapy and drug intervention), therapeutic approaches for regulating Treg-related signaling pathways, combined treatment strategies and targeted delivery systems, and clarify their therapeutic effects, experimental evidence and technical limitations (Table 3 and Table 4).

### 5.1. Targeted Interventions to Enhance Treg Cell Function

#### 5.1.1. Cell Therapy

Cell therapy is one of the important targeted intervention measures to enhance the function of Treg cells, which mainly includes Treg cell adoptive transfer and other cell-based therapies that can induce the generation and functional enhancement of Treg cells. Treg cell adoptive transfer has shown good therapeutic effects in a variety of inflammatory eye diseases such as corneal transplantation rejection, EAU and corneal mechanical injury, and local administration (subconjunctival injection, anterior chamber injection) has a better therapeutic effect than systemic administration [3,48,71,74]; A2Ar-dependent Treg subset adoptive transfer can reduce the recurrence rate of EAU and reduce ocular inflammatory infiltration [50]; mesenchymal stem cell (MSC) transplantation, human amniotic epithelial cell (hAEC) transplantation and IL-35+ Bregs adoptive transfer can induce the generation of antigen-specific Treg cells and alleviate ocular inflammation [14]; hUC-MSC-sEVs treatment can increase the proportion of Treg cells in the lacrimal gland and improve the secretion function of the lacrimal gland in autoimmune dacryoadenitis [6]; iMSC transplantation can promote the differentiation of Treg cells and protect retinal function in retinal I/R injury [10]; MDSCs adoptive transfer can enhance the function of Treg cells and alleviate dry eye symptoms [42]; and hUCMSC transplantation can increase the proportion of Treg cells and reduce conjunctival inflammation in allergic conjunctivitis [46]. Based on current evidence, MSCs and MSC-derived products hold the greatest therapeutic potential for: (1) Autoimmune ocular diseases (e.g., autoimmune uveitis, Sjögren’s syndrome) due to their potent immunomodulatory capacity; (2) severe corneal inflammation and high-risk corneal transplant rejection, where local administration can create a tolerogenic microenvironment; (3) retinal ischemic injuries (e.g., in glaucoma models), leveraging their neuroprotective and anti-inflammatory properties; and (4) severe immune-mediated dry eye disease, aiming to restore lacrimal gland function and ocular surface homeostasis.

#### 5.1.2. Pharmacological Interventions

The drug intervention strategies for enhancing the function of Treg cells are complex and diverse. This study classifies them into small-molecule drugs, plant extracts, and biological agents, clarifying the usage methods, action signaling pathways, experimental models, and anti-inflammatory effects of each drug, and providing candidate solutions for the development of drugs targeting Treg cells for inflammatory eye diseases. Small-molecule drugs such as CsA, VEGFR1R2Trap, Apumilast, AS101, low-dose IL-2 and Rac1 inhibitors can regulate the number and function of Treg cells by targeting specific signal pathways [1,2,14,17,36,64]; plant extracts such as sinomenine and Yiqi Jiedu Prescription (YQJD) can restore the balance of Th17/Treg cells by activating or inhibiting signal pathways [22,65]; and biological agents such as anti-IL-6r antibody and IL-10 monoclonal antibody can enhance the function of Treg cells or neutralize the cytokines secreted by Treg cells to play an anti-inflammatory role [36,40]; in addition, progesterone, melatonin and SAHA can also enhance the function of Treg cells by regulating epigenetic modifications and chemokine receptor-mediated homing [35,41,55].

### 5.2. Therapeutic Approaches for Regulating Treg-Related Signaling Pathways

#### 5.2.1. Targeting the P2X7 Receptor

Targeting the P2X7 receptor is an important therapeutic approach for regulating Treg-related signaling pathways, which mainly include P2X7 receptor antagonists and P2X7 gene knockout. oxATP, a P2X7 receptor antagonist, can inhibit the activation of P2X7 receptor, block the ERK1/2 and NF-κB signaling pathways, restore the proportion of Treg cells and the secretion of IL-10 and TGF-β, and alleviate retinal inflammatory infiltration [73]; P2X7 gene knockout can block the activation of P2X7 in macrophages and microglia, reduce the release of IL-1β, enhance the inhibitory effect of Treg cells on Th17 cells, and reduce retinal damage [39]; unspecified P2X7 receptor antagonists can block the P2X7 receptor-mediated suppression of Treg cell function, enhance the secretion of anti-inflammatory cytokines by Treg cells, and increase the survival rate of retinal ganglion cells [80].

#### 5.2.2. Targeting the Cytokine Network

As shown in Table 3, this study systematically reviews various intervention strategies targeting the cytokine network to regulate Treg cells, including the use of cytokine monotherapy and the combination of neutralizing antibodies, and clarifies the dose combinations, effects, and experimental models of each approach, providing strategic references for precise immune regulation in inflammatory eye diseases. Low-dose IL-2 can expand Treg cells and enhance their suppressive function [1]; IL-35+ Bregs exosomes can stimulate the secretion of IL-10 and IL-35 by Treg cells to alleviate EAU [14]; anti-IL-6r antibody can reduce the Th17 phenotype and increase the number of Treg cells [14]; IL-10 neutralizing antibody combined with TGF-β1 neutralizing antibody can synergistically inhibit the pro-angiogenic function of Treg cells [36]; and SF@DEX nanoparticles can regulate the balance of the IL-17A/IL-10/TGF-β1 signaling axis and enhance the immunosuppressive function of Treg cells [59].

#### 5.2.3. Comparative Analysis: Cell Therapy vs. P2X7R Antagonism

Although both Treg adoptive transfer (a form of cell therapy) and P2X7 receptor (P2X7R) antagonism can alleviate retinal inflammation, their mechanisms and ideal applications differ fundamentally. Mechanistically, Treg adoptive transfer is a “replacement” or “addition” strategy that directly supplies a large number of functional suppressor cells to actively impose immune suppression. In contrast, P2X7R antagonism is a “modulation” or “de-repression” strategy. It primarily targets innate immune cells (e.g., macrophages/microglia), inhibiting their release of pro-inflammatory cytokines like IL-1β. This action removes the suppressive pressure of the inflammatory microenvironment on endogenous Tregs, thereby indirectly restoring their function. Regarding application scenarios, cell therapy may be preferable for acute, severe inflammatory episodes (e.g., fulminant uveitis) where rapid, potent immunosuppression is needed. P2X7R antagonists might be better suited for chronic, low-grade inflammation or as preventive/maintenance therapy, where the goal is to subtly adjust the microenvironment to preserve endogenous immune regulation with potentially fewer side effects.

### 5.3. Combined Treatment Strategy

#### 5.3.1. Immunomodulation + Anti-Inflammatory Therapy

Combined treatment of “immunomodulation + anti-inflammatory therapy” is the development trend of Treg cell-based therapeutic strategies for inflammatory eye diseases, which can enhance the anti-inflammatory effect and reduce the side effects of single drugs by the synergistic effect of different therapies. CsA combined with topical/systemic steroids can synergistically inhibit inflammation and rescue steroid-resistant cases [2]; low-dose IL-2 combined with immunomodulators can specifically expand Treg cells and avoid systemic immunosuppression [14]; MDSCs combined with topical anti-inflammatory drugs can enhance the function of Treg cells and rapidly control ocular surface inflammation [42]; anti-IL-6 antibody combined with ocular surface anti-inflammatory preparations can restore the function of Treg cells and improve the stability of the tear film [60]; UC-MSC lenses combined with low-dose corticosteroids can synergistically enhance immunosuppression and reduce the dosage and side effects of hormones [75]; SpantideI combined with Th17 pathway inhibitors can restore the function of Treg cells and directly inhibit the pathogenic Th17 response [25].

#### 5.3.2. Targeted Delivery Systems

The ocular targeted delivery system is an important technical support to enhance the efficacy of Treg-related drugs and reduce systemic side effects, which can solve the problems of poor ocular targeting, insufficient local drug concentration and short retention time of traditional drug delivery methods. A variety of targeted delivery systems such as nanocarriers, eye drop carriers, in situ gel carriers, silk fibroin nanoparticles (SFNPs), hyaluronic acid methylcellulose (HAMC), hUC-MSC-sEVs, HAMA soluble microneedle patches and silicone hydrogel lenses have been developed and applied in the delivery of Treg-related drugs and cells [6,17,30,41,59,65,75,78]. These delivery systems can significantly prolong the ocular retention time of drugs/cells, increase the local drug concentration in the eye, and improve the therapeutic effect of Treg-related interventions. Our research team believes that the optimization of ocular targeted delivery systems is the key to the clinical transformation of Treg cell-based therapeutic strategies, and the development of novel targeted delivery systems with good biocompatibility, high targeting and sustained release is the future research direction.

## 6. Discussion and Outlook

### 6.1. Core Regulatory Characteristics and Mechanism Correlation of Treg Cells

Our research team systematically summarizes the research progress of Treg cells in inflammatory eye diseases and finds that Treg cells, as the core regulator of ocular immune homeostasis, have universal and disease-specific regulatory characteristics in inflammatory eye diseases. The universal regulatory characteristics are reflected in that the functional impairment or quantitative reduction in Treg cells is a common pathological feature of various inflammatory eye diseases, and targeted intervention to enhance Treg cell function shows universal anti-inflammatory potential in various ocular inflammatory models; the disease-specific regulatory characteristics are reflected in that the regulatory mechanisms of Treg cells in different inflammatory eye diseases are different due to the different pathological characteristics and microenvironmental changes in different ocular tissues.

Among various inflammatory eye diseases, Tregs play the most significant and direct role in autoimmune diseases. The regulatory mechanisms of Treg cells in inflammatory eye diseases share three core commonalities: First, the immune-privileged microenvironment of the eye is the core supporting basis for the function of Treg cells. The low immunogenicity of the eye, the enrichment of anti-inflammatory cytokines and the physical isolation provided by the blood–eye barrier are the prerequisite for Treg cells to exert their immunosuppressive function [3,80]. TGF-β and RA from the aqueous humor and PD-L1 expressed by ocular resident cells induce the differentiation of Treg cells and stabilize the expression of Foxp3 [3,9]; while the disruption of the blood–eye barrier caused by various pathological factors leads to the infiltration of inflammatory cells and the enrichment of pro-inflammatory factors, which can induce Treg cells to transform into effector-like cells (ex-Tregs) and weaken their inhibitory function [10,24]. This pathological process is manifested in various inflammatory eye diseases such as corneal inflammation, uveitis and retinal diseases [10,11,62]. Second, the cross-regulatory network of signal pathways is the core internal mechanism for the function of Treg cells. The STAT family (STAT3/STAT5), PI3K/AKT, SIRT1, Rac1 and other signal pathways constitute the core network for regulating the function of Treg cells [12,33,35,55]. These pathways interact with each other in different inflammatory eye diseases and jointly regulate the balance between Treg and effector T cells [55,63]. Third, the bidirectional regulation of the cytokine network is the key external mechanism for the function of Treg cells. IL-10 and TGF-β, as the core anti-inflammatory cytokines secreted by Treg cells, play a crucial role in corneal transplant tolerance and retinal inflammation resolution [1,7,11], while pro-inflammatory factors such as IL-6 and TNF-α induce Treg cell functional defects by downregulating the expression of Foxp3 and CD25 [4,43,46]. This bidirectional regulation of the cytokine network is the key to maintaining the balance of Treg cell function in the ocular microenvironment.

### 6.2. Research Limitations

Although the regulatory role of Treg cells in inflammatory eye diseases has been widely confirmed, and Treg cell-based targeted therapeutic strategies have shown good anti-inflammatory potential in animal models, the current research still faces many deficiencies and limitations, which restrict the clinical translation and application of Treg cell-based therapies:

(1) Insufficient analysis of Treg cell heterogeneity: Treg cells exhibit significant subpopulation heterogeneity (such as CD25lo/hi, antigen-specific Treg, effector memory Treg), and the functional differences among different subpopulations in different inflammatory eye diseases have not been fully clarified [24,31]. For instance, CD25loTreg cells are prone to transform into ex-Treg cells and promote inflammation after HSV-1 infection [24], while antigen-specific Treg cells show stronger targeting in the treatment of uveitis [3]. However, there is currently a lack of systematic comparative studies on the phenotypes and functions of Treg subpopulations in different eye diseases, which makes it impossible to realize the precise regulation of Treg subpopulations.
(2) Disconnection between basic research and clinical translation: Most existing studies are based on animal models (such as mouse EAU models, corneal transplantation models, OIR models), and human clinical data is limited [3,14]. For example, low-dose IL-2 can effectively expand Treg cells and alleviate retinal inflammation in animal models [1], but its efficacy and safety in human non-infectious uveitis still require large-scale clinical trials for verification; Treg cell adoptive transfer shows good anti-inflammatory effects in animal experiments [10,54], but faces technical bottlenecks such as cell source, in vitro expansion efficiency, and in vivo homing specificity in clinical application [3,5]. In addition, the lack of standardized clinical detection methods for Treg cells also restricts the clinical application of Treg cell-based therapies.
(3) Insufficient research on the mechanisms of some diseases: The regulatory role and mechanism of Treg cells in some inflammatory eye diseases such as endophthalmitis and retinal chorioretinitis remain unclear. Existing research mainly focuses on corneal inflammation, uveitis and dry eye [39,74], lacking original experimental evidence for these diseases, which limits the application scope of Treg-related treatment strategies.
(4) Imperfect targeted delivery system: The current drug interventions (such as sCD83, NAD+ and IL-2) are mostly administered systemically or delivered locally in a simple manner, which leads to problems such as poor ocular targeting, insufficient local drug concentration, and systemic side effects [7,13,29]. Although delivery carriers such as liposomes, nanoparticles, and hydrogels have shown potential [30,59,71], it is still necessary to optimize the particle size, biocompatibility, and drug release kinetics of the carriers to enhance the targeting and efficacy of Treg-related interventions.

### 6.3. Future Research Directions

In view of the current research limitations, our research team proposes the following future research directions for Treg cell-based research and treatment of inflammatory eye diseases, which aim to solve the current technical bottlenecks and promote the clinical translation and application of Treg cell-based immunomodulatory therapies:

(1) Systematically analyze the disease-specific functions of Treg subgroups by multi-omics technologies: Utilize single-cell RNA sequencing, flow cytometry, spatial transcriptomics and other multi-omics technologies to systematically analyze the phenotypic characteristics (surface markers, transcription factor expression, metabolic characteristics) of and functional differences in Treg subgroups in different inflammatory eye diseases, clarify the regulatory roles of antigen-specific Tregs, effector memory Tregs and other subgroups in different ocular inflammatory pathologies, and screen the specific surface markers of functional Treg subgroups. On this basis, we aim to develop precise targeted intervention strategies for Treg subgroups, realize the precise regulation of Treg cells, and improve the therapeutic specificity and efficiency.
(2) Optimize Treg-related targeted delivery systems based on ocular tissue specificity: Based on the anatomical characteristics and tissue specificity of different ocular tissues (cornea, retina, uvea, etc.), develop novel ocular targeted delivery carriers with good biocompatibility, high targeting and sustained release, such as cornea-penetrating nanoparticles, retinal-targeted liposomes, injectable hydrogels and biodegradable microneedle patches. Realize the local efficient delivery of Treg cells and related drugs, increase the concentration of drugs/cells in the target ocular tissue, prolong the retention time, and reduce systemic side effects. In addition, we aim to develop personalized targeted delivery systems according to the different pathological characteristics of patients, further improve the therapeutic effect.
(3) Accelerate the clinical translational research of Treg cell-based therapeutic strategies: Carry out small-sample clinical trials to evaluate the safety, efficacy, and optimal dosage of Treg-related interventions (e.g., low-dose IL-2, Treg adoptive transfer, and STAT3 inhibitors) in patients with human inflammatory eye diseases such as non-infectious uveitis and high-risk corneal transplant rejection. Establish a standardized clinical sample bank to systematically collect clinical data (such as disease severity, treatment response, and prognosis) and biological samples (peripheral blood, aqueous humor, vitreous fluid) from patients, analyze the correlation between Treg cell proportion, functional status, and disease progression/prognosis, and screen potential Treg-related biomarkers for disease diagnosis, efficacy evaluation, and prognosis prediction [37,80]. Strengthen the cooperation between basic research institutions and clinical institutions to promote the two-way translation of basic research results and clinical needs.
(4) Develop novel regulatory targets based on multi-omics technologies: Combine genomics, transcriptomics, proteomics, metabolomics, and epigenomics technologies to comprehensively analyze the molecular mechanisms of Treg cell function regulation in inflammatory eye diseases. Focus on exploring the epigenetic regulatory mechanisms (such as TSDR methylation [24], TET2/NT5E axis [35]) and metabolic reprogramming characteristics (such as glycolysis, oxidative phosphorylation, amino acid metabolism) of Treg cells in different ocular inflammatory microenvironments and identify new key regulatory molecules and signaling pathways (such as PIM1, Rac1 [33,63]). On this basis, we aim to develop novel targeted drugs with higher specificity and stronger efficacy, and provide new ideas and targets for the precise treatment of inflammatory eye diseases.
(5) Optimize combined treatment strategies based on precision medicine: According to the different pathological types, severity degrees, and patient individual characteristics of inflammatory eye diseases, develop personalized combined treatment strategies. Explore the optimal combination of “immune regulation + anti-inflammatory treatment” (such as Treg adoptive transfer + local anti-inflammatory drugs, IL-6 antagonists + ocular surface anti-inflammatory preparations [60,63]), and achieve synergistic enhancement of Treg function, rapid control of acute inflammation, reducing the dose-dependent toxicity of single treatment, and achieving both symptomatic and root-cause treatment [2,42].

In summary, Treg cells, as the core regulators of ocular immune homeostasis, play a crucial role in the pathogenesis of inflammatory eye diseases. Targeted interventions based on Treg cells offer new therapeutic approaches for these conditions. In the future, it is necessary to further elucidate the disease-specific regulatory mechanisms of Treg cells, overcome technical bottlenecks, promote the translation of basic research into clinical applications, and ultimately develop safe and effective novel immunomodulatory therapies to improve the visual prognosis of patients with inflammatory eye diseases.

## Figures and Tables

**Table 1:** Distribution and Characteristics of Treg Cells in Ocular Tissues.

Ocular Tissues/Sites	Distribution Characteristics	Associated Inflammatory Eye Diseases	Core Regulatory Mechanisms	Detection Methods	References
Cornea	Steady state: Anterior stroma near epithelial layer (peripheral density > central); Inflammation: Infiltration in graft/host stroma; HSV-1 infection: Increased CD25loTreg; FECD: Negatively correlated with TRPV1	Corneal transplant rejection, HSK, Aspergillus fumigatus keratitis, FECD, corneal nerve injury-related inflammation	1. Corneal transplant rejection: Inhibit (lymph)angiogenesis, secrete IL-10 to protect CEnCs; 2. HSK: CD25loTreg → ex-Treg, pDCs-TLR9-IFN-α maintains Treg stability; 3. Others: CD3ε upregulates IL-10, TRPV1 regulates Treg function, substance P downregulates Treg CD103	Flow cytometry, histological analysis, immunohistochemistry, qPCR	[16,17,21,22,23,24,25,26,27,28,29]
Retina	Steady state: Few in blood vessels and GCL; Inflammation: Enriched in neovascularization/inflammatory sites, co-localized with Th17; Melatonin treatment: 6-fold increase in middle/deep retina	EAU, OIR, AMD, retinal I/R injury	1. EAU: Th17/Treg balance, PIM1/AKT/FOXO1 pathway, A2Ar-dependent Treg homing; 2. OIR: Low-dose IL-2 restores Treg function, CTLA-4 inhibits microglia; 3. AMD: Rac1 signaling pathway, TET2/NT5E-CCR4 axis; 4. Retinal I/R injury: iMSC mitochondrial transport promotes Treg differentiation	Flow cytometry, immunofluorescence, histological analysis	[2,10,11,14,30,31,32,33,34,35,36]
Uvea	Steady state: Few distribution; Inflammation: Increased recruitment; Uveal melanoma: Increased Foxp3^+^ Treg; Tuberculous uveitis: Decreased Treg infiltration	Uveitis (EAU, tuberculous, chronic autoimmune), uveal melanoma-associated inflammation	1. Tuberculous uveitis: Decreased Treg frequency, downregulated TGF-β/IL-2Rα, weakened inhibition on Th1/Th17; 2. Uveal melanoma: IL-6/IP-10 regulates Treg infiltration	Flow cytometry, histological analysis, clinical sample detection	[37,38,39,40]
Lacrimal Gland	Steady state: CD4^+^ CD25^+^ Foxp3^+^ Treg exists; CD25KO mice: Significantly reduced Treg; Aged mice: Increased Treg proportion with activation markers; Autoimmune dacryoadenitis: Decreased Treg proportion	Dry eye, immune aging, autoimmune dacryoadenitis	1. Autoimmune dacryoadenitis: Downregulated Foxp3/Nurr1, Th17/Treg imbalance, TNF-α/IL-1β inhibits Treg; 2. Dry eye: Age-related Treg functional decline	Flow cytometry, histological analysis, qPCR	[6,20,41,42]
Conjunctiva	Steady state: Detectable Treg in peripheral blood/tear of healthy donors; Inflammation: Dynamic migration to lesions; Dry eye (SS-related): Higher Treg proportion; Conjunctival squamous cell carcinoma: Foxp3^+^ Treg in stroma/tumor, higher in Tadv than Tis group	Allergic conjunctivitis, dry eye, conjunctival squamous cell carcinoma	1. Allergic conjunctivitis: SLAM-mediated Treg/effector T-cell imbalance, miR-146a regulates NF-κB, α-MSH induces Treg; 2. Conjunctival squamous cell carcinoma: Foxp3/CXCR4 axis mediates Treg infiltration	Flow cytometry, immunohistochemistry, clinical sample detection	[15,25,43,44]
Aqueous Humor	Corneal transplantation: Detectable CD4^+^ Foxp3^+^ Treg; Treg-related cytokines (IL-10, TGF-β) detectable	Corneal graft rejection, uveitis	1. Corneal transplant tolerance: ACAID-induced antigen-specific Treg; 2. Uveitis: Cytokine network regulates Treg function	Flow cytometry, cytokine detection	[3,37,38]
Iris-Ciliary Body	Enriched CD4^+^ Foxp3^+^ Treg; ACAID-mediated immune tolerance induction	Corneal transplant tolerance, uveitis	ACAID: APCs capture antigens to induce Treg, maintaining immune homeostasis	Flow cytometry, histological analysis	[3,18]
Vitreous Body	Inflammation: Increased infiltration; Human vitreous: CD3^+^ CD4^+^ CD25^+^ CD127−Treg exists; B-VRL: Significantly decreased Treg	Uveitis, B-VRL	Uveitis: Treg functional impairment contributes to chronic inflammation	Flow cytometry, clinical sample detection	[17,37,45]
Draining Lymph Nodes (Neck /Submandibular)	Main site of Treg proliferation/differentiation; Inflammation (corneal transplantation, allergic conjunctivitis, EAU, dry eye): Significantly increased Treg proportion	Corneal graft rejection, allergic conjunctivitis, EAU, dry eye	1. Corneal transplantation: Treg expansion inhibits allogeneic immune response; 2. Dry eye: SP-NK-1R signaling impairs Treg function	Flow cytometry, qPCR	[2,3,4,9,17,20,25,33,39,46,47]
Lens	Epithelial cells: Resident immune cells with Treg characteristics; Cataract surgery: Activated and aggregated at injury sites	Inflammation after cataract surgery	Trauma-induced Treg activation contributes to tissue repair	Histological analysis	[18]

Note: A2Ar: Adenosine A2A receptor; ACAID: Anterior chamber-associated immune deviation; AMD: Age-related macular degeneration; APCs: Antigen-presenting cells; B-VRL: B-cell vitreoretinal lymphoma; CD103: Cluster of differentiation 103; CD127: Cluster of differentiation 127; CD25: Cluster of differentiation 25; CD25KO: CD25 knockout; CD25lo: CD25 low; CD3ε: CD3 epsilon; CD4: Cluster of differentiation 4; CEnCs: Corneal endothelial cells; CTLA-4: Cytotoxic T-lymphocyte associated protein 4; CXCR4: C-X-C chemokine receptor type 4; EAU: Experimental autoimmune uveitis; FECD: Fuchs’ endothelial corneal dystrophy; Foxp3: Forkhead box P3; GCL: Ganglion cell layer; HSK: Herpes simplex keratitis; HSV-1: Herpes simplex virus type 1; I/R: Ischemia/reperfusion; IFN-α: Interferon-alpha; IL-10: Interleukin-10; IL-2: Interleukin-2; IL-6/IP-10: Interleukin-6/interferon gamma-induced protein 10; iMSC: Induced mesenchymal stem cells; miR-146a: MicroRNA-146a; NF-κB: Nuclear factor kappa-B; Nurr1: Nuclear receptor related 1; OIR: Oxygen-induced retinopathy; pDCs: Plasmacytoid dendritic cells; PIM1/AKT/FOXO1: PIM1 proto-oncogene, serine/threonine kinase/protein kinase B/forkhead box O1; qPCR: Quantitative polymerase chain reaction; Rac1: Ras-related C3 botulinum toxin substrate 1; SLAM: Signaling lymphocytic activation molecule; SP-NK-1R: Substance P - neurokinin-1 receptor; TET2/NT5E-CCR4: TET methylcytosine dioxygenase 2/5′-nucleotidase ecto - C-C chemokine receptor type 4; TGF-β: Transforming growth factor-beta; TGF-β/IL-2Rα: Transforming growth factor-beta/interleukin-2 receptor alpha; Th1/Th17: T helper type 1/T helper type 17; Th17: T helper type 17; Th17/Treg: T helper type 17/regulatory T cell; TLR9: Toll-like receptor 9; TNF-α/IL-1β: Tumor necrosis factor-alpha/interleukin-1 beta; TRPV1: Transient receptor potential vanilloid 1; α-MSH: Alpha-melanocyte stimulating hormone.

**Table 2:** Key Factors Modulating Treg Cell Function in Inflammatory Eye Diseases.

Types of Factors	Specific Factors	Mechanism of Action	Related Diseases	References
Pharmacological Interventions	CsA	Enhance IL-2 signaling pathway, reverse Treg inflammatory phenotype, promote Treg proliferation	Autoimmune uveitis	[2]
	VEGFR1R2Trap	Increase Treg frequency in draining lymph nodes, inhibit DCs activation	Corneal transplant rejection	[17]
	Apumilast	Block PI3K/AKT pathway, increase Treg numbers	EAU	[14]
	AS101	Inhibit phosphorylation of AKT and STAT3/4, promote naive T cells to differentiate into Treg	EAU	[14,61]
	MTX	Inhibit purine synthesis, regulate Treg/Teff balance	EAU	[62]
	MMF	Inhibit guanine nucleotide synthesis, promote Treg activation	EAU	[62]
	Rac1 inhibitors (1A-116, NSC23766)	Inhibit Rac1 signaling pathway	AMD, EAU	[36,63]
	Low-dose IL-2	Bind IL-2 receptor, promote Treg proliferation, upregulate inhibitory molecules (CTLA-4, PD-1, TIGIT)	Diabetic retinopathy, OIR, corneal transplantation	[1,64]
	Sinomenine	Inhibit PI3K/AKT and NF-κB signaling pathways, restore Th17/Treg balance	EAU	[65]
	Yiqi Jiedu Prescription (YQJD)	Activate STAT5 signaling pathway	Recurrent HSK	[22]
	Progesterone	Upregulate Treg functional molecules, inhibit Id2/Pim1 axis	EAU	[55]
	Melatonin	Activate TET2/NT5E axis, promote CCR4-mediated Treg recruitment	AMD	[35]
Signaling Pathways	PI3K/AKT pathway	Regulate Treg/Th17 balance, pathway blockade enhances Treg function	EAU, uveitis	[14,65]
	STAT pathway	STAT5 phosphorylation induces Treg expansion; STAT1/3 inhibition increases Treg frequency; STAT3 maintains Treg quiescence	EAU, uveitis	[14,49]
	IL-2 signaling pathway	Maintain Treg survival and homeostasis, enhance immunosuppressive capacity	Autoimmune uveitis	[2]
	NF-κB pathway	Affect Treg immunosuppressive activity, promote pro-inflammatory factor secretion, weaken Treg function	Uveitis	[65]
	SIRT1 pathway	Activate to enhance Treg function, regulate Th1/Th17/Tregs balance	Optic neuritis	[13]
	A2Ar pathway	Regulate homing and function of Treg subsets	EAU	[50]
	PIM1/AKT/FOXO1 pathway	Inhibit Treg activation	EAU	[33]
	Rac1/Id2/Pim1 axis	Promote Th17 pathogenicity, reduce Treg proportion	EAU	[63]
Cytokines	IL-6	Induce Treg dysfunction, downregulate Foxp3/CD25, promote Treg → Th17 transformation	Dry eye, uveitis, myopia	[14,16,37,43,60]
	IL-10	Enhance Treg inhibitory activity, inhibit effector T cell activation	Uveitis, HSK, dry eye	[2,14,22,65,66,67]
	TNF-α	Inhibit Treg proliferation and function, aggravate immune imbalance	Uveitis, dry eye, autoimmune dacryoadenitis	[6,14,16,65,66]
	TGF-β	Induce Treg differentiation, enhance immunosuppressive function, maintain Treg stability	Multiple inflammatory eye diseases	[3,8,14,31]
	IL-12	Activate STAT4 pathway, promote Treg → Th1-like cell transformation	HSK	[24]
	IL-17	Antagonize Treg function, weaken immunosuppressive effect	Uveitis	[2,33]
	α-MSH	Induce Treg differentiation, inhibit TLR4 activation	Allergic conjunctivitis	[43]
Microbiome	Gut microbiome disturbance	Affect butyrate production, regulate Treg differentiation and function	EAU	[14]
	Vancomycin/metronidazole	Increase Treg number in retina and lymph nodes, reduce EAU severity	EAU	[14]
	Akkermansia	Inhibit inflammation, inversely correlated with intraocular TNF-α^+^ T cells	EAU	[62]
	LachnospiraceaeNK4A136	Promote Treg activation	EAU	[62]
Inflammatory Microenvironment	IL-6, TNF-α	Promote Treg→Th17 phenotype transformation, weaken immunosuppressive function	Uveitis, myopia	[14,16]
	IL-17 signaling pathway	Antagonize Treg function	Autoimmune uveitis	[2]
	Dry stress	Cause Treg dysfunction, reduce inhibitory capacity	Dry eye	[25,60]
	Pro-inflammatory factor enrichment	Induce Treg phenotype switching, loss of immunosuppressive function	Multiple inflammatory eye diseases	[18,24]
Cell–Cell Interaction	DC phenotypes	CD200R+ DCs induce Treg proliferation and enhance immune tolerance; Tolerogenic DCs promote Treg differentiation	Corneal graft rejection, EAU	[7,14,17]
	Effector T cells	Th1/Th17 cells antagonize Treg, affect inflammation outcome	Uveitis	[2,14]
	Macrophages	M2 macrophages secrete Arg1/IL-10 to promote Treg generation; M1 macrophages secrete TNF-α/IL-1β to inhibit Treg function	Autoimmune dacryoadenitis, AMD	[6,11,54]
	pDCs	Secrete IFN-α, maintain Treg stability	HSK	[23]
	B cells	STAT3 deficiency inhibits Treg development; CD80/CD86 expression regulates Treg function	EAU	[68]
Other Factors	Age	Aged Treg shows activated effector memory phenotype, decreased inhibitory function; non-Treg cell depletion	Dry eye, immune aging	[5,20]
	Antigen-specific stimulation	Retinal autoantigen (IRBP) and Mycobacterium tuberculosis antigen induce Treg multifunctional response	Uveitis	[66]
	TLR2 signaling pathway	Promote Treg proliferation and IL-10 secretion, enhance anti-angiogenic and anti-inflammatory functions	Corneal inflammation	[69]
	CTLA-4 molecule	Regulate tissue-specific Treg function; Splenic Treg requires CTLA-4, ocular Treg is CTLA-4-independent	EAU	[70]
	PD-1/PD-L1	PD-L1 binds to Treg surface PD-1, enhances Treg activity	EAU	[48,71]
	TIGIT	Positively correlated with Foxp3, enhances Treg inhibitory function on Th17 cells after stimulation	EAU	[50,72]
	CD25 molecules	CD25 deficiency leads to reduced Treg number and functional defects	Autoimmune dacryoadenitis	[5,6]
	Epigenetic modifications (TSDR methylation)	Demethylation maintains stable Foxp3 expression; Methylation leads to Treg functional instability	HSK, EAU	[24,31]
	P2X7 receptor	Macrophage P2X7 activation promotes IL-1β release, indirectly weakens Treg suppressive function	EAU	[39,73]
	SP-NK-1R signal	SP binds to Treg surface NK-1R, downregulates Foxp3 and CTLA-4 expression	Dry eye, corneal nerve injury	[9,25]

Note: Arg1: Arginase-1; CCR4: C-C chemokine receptor type 4; CsA: Cyclosporin A; DC: Dendritic cell; Id2: Inhibitor of DNA binding 2; IL-12: Interleukin-12; IL-17: Interleukin-17; MMF: Mycophenolate mofetil; MTX: Methotrexate; P2X7: P2X purinoceptor 7; PD-1: Programmed cell death protein 1; PD-L1: Programmed death-ligand 1; PI3K: Phosphatidylinositol 3-kinase; SIRT1: Sirtuin 1; STAT: Signal transducer and activator of transcription; STAT1: Signal transducer and activator of transcription 1; STAT3: Signal transducer and activator of transcription 3; STAT4: Signal transducer and activator of transcription 4; STAT5: Signal transducer and activator of transcription 5; TIGIT: T cell immunoreceptor with Ig and ITIM domains; TLR2: Toll-like receptor 2; TSDR: Treg-specific demethylated region.

**Table 3:** Treg cell-based therapeutic strategies for inflammatory eye diseases (including cell therapy and drug intervention).

Therapeutic Type	Specific Strategy	Route of Administration	Target Pathway/Mechanism	Experimental Model	Efficacy Outcomes	Technical Limitations	References
Cell Therapy	MSC Transplantation	Intraperitoneal injection	Induce antigen-specific Treg generation	EAU mouse model	Prevent EAU recurrence, long-term Treg survival	May exert pro-inflammatory effects in inflammatory microenvironment	[14]
	hAEC Transplantation	Subretinal injection	Increase Treg/Th17 ratio	EAU rat model	Reduce pathological score	Therapeutic effect depends on administration time	[14]
	IL-35+ Bregs Adoptive Transfer	Intraperitoneal injection	Promote Treg expansion, inhibit Th1/Th17 response	EAU mouse model	Alleviate inflammation severity	Need to generate autologous Bregs in vitro	[14]
	Treg Adoptive Transfer	Subconjunctival injection, anterior chamber injection, tail vein injection	Directly supplement functional Treg, inhibit immune response	Mouse corneal transplantation model, EAU mouse model, corneal mechanical injury model	Improve graft survival rate, reduce rejection index, accelerate corneal wound healing, reduce retinal inflammation	Difficulty in preparing antigen-specific Treg; low in vitro expansion efficiency; poor ocular targeting of systemic administration	[3,48,71,74]
	A2Ar-Dependent Treg Subset Adoptive Transfer	Tail vein injection	Regulate Treg homing and function	EAU mouse model	Reduce disease recurrence rate, alleviate ocular inflammatory infiltration	Complex and expensive clinical-grade Treg subset sorting technology	[50]
	hUC-MSC-sEVs Treatment	Subconjunctival injection	Promote M2 macrophage polarization, induce Treg via miR-100-5p	Rabbit model of autoimmune dacryoadenitis	Increase lacrimal gland Treg proportion, improve tear secretion, reduce inflammatory infiltration	Long-term efficacy needs verification; administration frequency needs optimization	[6]
	iMSC Transplantation	Intravitreal injection	Promote Treg differentiation via mitochondrial transport	Mouse retinal I/R injury model	Increase retinal Foxp3^+^ Treg number, improve b-wave amplitude	Unclear Treg source (local upregulation or peripheral recruitment); unelucidated downstream molecules	[10]
	MDSCs Adoptive Transfer	Subconjunctival injection	Enhance Treg functional stability via IL-10 secretion	Mouse dry eye model	Reduce corneal fluorescein staining score, enhance Treg function	Complex in vitro cell expansion technology; difficult clinical translation	[42]
	hUCMSCs Transplantation	Subconjunctival injection	Regulate T cell response, increase Treg proportion	Mouse experimental allergic conjunctivitis model	Reduce conjunctival inflammation, decrease Th2/Th17 ratio	Intravenous administration has no significant effect	[46]
Drug Intervention (Small Molecule Drugs)	CsA	Intraperitoneal injection (20 mg/kg/day for 2 weeks)	Enhance IL-2 signaling pathway, inhibit NF-κB pathway	EAU mouse model	Reduce Th1/Th17 ratio, increase Treg number, decrease clinical/histological scores	Systemic side effects with long-term use	[2]
	VEGFR1R2Trap Eye Drops	Topical (10 mg/mL, 3 times/day for 2 weeks)	Inhibit VEGF signaling pathway	Mouse corneal transplantation model	Increase Treg frequency in draining lymph nodes, improve graft survival	Local irritation may occur	[17]
	Apumilast	In vitro intervention + in vivo administration	Block PI3K/AKT/FOXO1 pathway	EAU mouse model	Reduce disease severity, increase Treg number, decrease Th17 cells	Potential gastrointestinal side effects	[14]
	AS101	In vitro (5 μg/mL) + intraperitoneal injection (27 μg/rat for 14 days)	Inhibit AKT, STAT3/4 phosphorylation	EAU mouse model	Promote naive T cell → Treg transformation, increase splenic Treg proportion	Need to optimize in vivo administration dose	[14,61]
	Low-dose IL-2	Intraperitoneal injection (25,000 units/dose)	Bind IL-2 receptor, promote Treg proliferation	Diabetic retinopathy mice, OIR mice	Restore Treg/CD8^+^ T ratio, reduce vascular injury and neovascularization	Risk of activating effector T cells at high doses	[1,64]
	1A-116	Intraperitoneal injection (3 mg/kg, twice a week)	Inhibit Rac1 signaling pathway	Laser-induced AMD mouse model	Reduce choroidal neovascularization area and microvessel density	Need to improve ocular targeting	[36]
Drug Intervention (Plant Extracts/Prescriptions)	Sinomenine	Oral (25 mg/kg, 50 mg/kg for 12 days)	Inhibit PI3K/AKT and NF-κB signaling pathways	EAU rat model	Decrease ocular inflammation score, increase IL-10 level	Low bioavailability	[65]
	Yiqi Jiedu Prescription (YQJD)	Gavage (1100 mg/mL)	Activate STAT5 signaling pathway	Mice with recurrent HSK	Reduce corneal injury score, increase Treg proportion and IL-10/TGF-β levels	Complex composition, unclear active ingredients	[22]
Drug Intervention (Biological Agents)	Anti-IL-6R Antibody (Tocilizumab)	Clinical routine dose	Inhibit IL-6 signaling pathway	Uveitis patients	Enhance Treg function, improve anatomical outcome of macular edema	Risk of infection with long-term use	[40]
	IL-10 Monoclonal Antibody (JES5-2A5)	Intraperitoneal injection (1 mg/kg, twice a week)	Neutralize Treg-derived IL-10	Laser-induced AMD mouse model	Reduce intraocular VEGFA/Ang2 levels, inhibit choroidal neovascularization	May affect normal immune tolerance	[36]
Drug Intervention (Others)	Progesterone	Intraperitoneal injection (50 mg/kg, days 2–14 after immunization)	Inhibit Id2/Pim1 axis, upregulate Treg functional molecules	EAU mouse model	Decrease clinical/histological scores, reduce Th17 cell proportion	Hormonal side effects (e.g., irregular menstruation)	[55]
	Melatonin	Intraperitoneal injection (10 mg/kg, twice a week for 4 weeks)	Activate TET2/NT5E axis, promote CCR4-mediated Treg recruitment	AMD mouse model	Increase retinal thickness, decrease apoptosis, increase M2 macrophage proportion	Need to optimize administration frequency	[35]
	SAHA	Lacrimal gland injection (10 mg/mL, PLGA microsphere-loaded)	Promote Foxp3 acetylation	Concanavalin A-induced DED mice	Restore tear secretion, reduce pro-inflammatory factors	Invasive administration	[41]
	miR-146a Mimics	Tail vein injection (lentiviral vector packaging)	Inhibit NF-κB signaling pathway	Allergic conjunctivitis mice	Decrease IgE, IL-5/IL-13 levels and eosinophil infiltration	Potential off-target effects	[58]

Note: 1A-116: 1A-116 (a Rac1 inhibitor); AS101: Ammonium trichloro(dioxoethylene-O,O′)tellurate; hAECs: Human amniotic epithelial cells; hUC-MSC-sEVs: Human umbilical cord mesenchymal stem cell-derived small extracellular vesicles; hUCMSCs: Human umbilical cord mesenchymal stem cells; IL-35: Interleukin-35; Low-dose IL-2: Low-dose interleukin-2; MDSCs: Myeloid-derived suppressor cells; MSCs: Mesenchymal stem cells; PLGA: Poly(lactic-co-glycolic acid); SAHA: Suberoylanilide hydroxamic acid; VEGFR1R2Trap: Vascular endothelial growth factor receptor 1 and 2 Trap.

**Table 4:** Combined treatment and targeted delivery systems for treg cell-based therapy.

Therapeutic Category	Specific Strategy	Key Components/Carriers	Experimental Model	Therapeutic Advantages	Technical Parameters	References
Combined Treatment (Immunomodulation + Anti-Inflammatory Therapy)	CsA + Topical/Systemic Steroids	CsA + Glucocorticoids	EAU mouse model + clinical patients	Synergistically inhibit inflammation; rescue steroid-resistant cases	No serious complications	[2]
	Low-dose IL-2 + Immunomodulators	IL-2 + Immunomodulators	Behcet’s disease patients	Specifically expand Treg; avoid systemic immunosuppression	No obvious adverse reactions	[14]
	MDSCs + Topical Anti-Inflammatory Drugs	MDSCs + Ocular surface anti-inflammatory preparations	Mouse dry eye model (in vitro validation)	Enhance Treg function; rapidly control ocular surface inflammation; protect corneal epithelium	No reported complications	[42]
	Anti-IL-6 Antibody + Ocular Surface Anti-Inflammatory Preparations	Anti-IL-6 antibody + Ocular surface anti-inflammatory drugs	Mouse dry eye model (in vitro validation)	Restore Treg function; reduce corneal fluorescein staining; improve tear film stability	No reported complications	[60]
	UC-MSC Lenses + Low-dose Corticosteroids	UC-MSC-loaded silicone hydrogel lenses + Low-dose glucocorticoids	Rabbit model of high-risk corneal transplant rejection	Synergistically enhance immunosuppression; reduce hormone dosage and side effects	Reduce complication rate by >30%	[75]
	Spantide I + Th17 Pathway Inhibitors	NK-1R antagonist + Th17 pathway inhibitors	Mouse DED model	Restore Treg function; directly inhibit pathogenic Th17 response	Significantly reduce corneal inflammation and epithelial injury	[25]
	Berberine + Dexamethasone	Berberine + Dexamethasone	EAU rats	Sustained drug release; prolong anti-inflammatory effect; reduce single-drug dose dependence	No obvious complications	[76]
Targeted Delivery Systems	Nanocarriers	Everolimus-loaded nanocarriers	EAU mouse model	Extend ocular retention time; improve drug bioavailability	Particle size: 50–100 nm; Ocular retention: Extended to 72 h	[77]
	Eye Drop Carriers	VEGFR1R2Trap-loaded eye drops	Mouse corneal transplantation model	Non-invasive administration; maintain local drug concentration	Detectable for 14 days after transplantation	[17]
	Silk Fibroin Nanoparticles (SFNPs)	Dexamethasone-loaded SFNPs	SD rat retinal detachment model	Sustained drug release; increase local drug concentration	Particle size: 100.22 ± 2.41 nm; Release duration: 12 h	[59]
	Hyaluronan Methylcellulose (HAMC)	Treg-loaded HAMC	EAU mouse model	Enhance Treg retention and survival in the eye	Retention time: 24 h (high proportion of Treg detectable)	[30]
	hUC-MSC-sEVs	miR-100-5p-loaded hUC-MSC-sEVs	Rabbit model of autoimmune dacryoadenitis	Targeted delivery to lacrimal gland; enhance miR-100-5p expression	Particle size: 50–150 nm (peak 113 nm); Retention time: ≥7 days	[6]
	HAMA Soluble Microneedle Patch	PKHB1 peptide-loaded microneedle patch	HSK mouse model	Enhance local drug concentration; minimally invasive administration	Microneedle height: 200 μm; Base diameter: 100 μm; Retention time: 12 h	[78]
	Collagen Scaffold Carriers	Treg-loaded collagen scaffolds	Mouse corneal alkali burn model	Enhance Treg local retention; improve cell survival rate	Retention time: 48 h; Cell survival rate increased by 40%	[54]
	Mesoporous Silica Nanoparticles (MSNs) + Thermal Gels	Berberine + Dexamethasone-loaded MSNs + thermal gels	EAU rats	Prolong drug release; increase intraocular drug concentration	Particle size: 33.5 ± 5.2 nm; Retention time: Up to 4 weeks	[76]
	Polylactic Acid-Co-Glycolic Acid (PLGA) Microspheres	SAHA-loaded PLGA microspheres	Concanavalin A-induced DED mice	Sustained local drug release; avoids systemic side effects of invasive administration	Particle size: ~17 μm; In vitro cumulative release: ~50 ng/mg over 5–6 days	[41]
	Poly(Lactic Acid-Caprolactone) Micromembrane	Tacrolimus-loaded micromembrane	Allergic conjunctivitis model	Inhibits eosinophil infiltration; improves ocular surface immune microenvironment	No quantified concentration data; biocompatible with ocular surface	[79]
	Liposome Carriers	PEDF-loaded liposomes	Dry eye model	Enhances local bioavailability of PEDF; promotes Treg suppressive phenotype	Particle size: 80–120 nm; Ocular retention: Extended to 48 h; Concentration enhancement: 2.8-fold	[29]
	Extracellular Vesicles/mPEG-hexPLA Nanocarriers	Everolimus-loaded nanocarriers	EAU mouse model	Prolongs drug residence time; reduces systemic immunosuppression	Particle size: 50–100 nm; Ocular retention: Extended to 72 h; Concentration enhancement: 3.2-fold	[77]
	In Situ Gel Carriers	Sinomenine-loaded in situ gel	EAU rat model	Extends ocular retention time; improves bioavailability of lipophilic drugs	No quantified particle size; sustained release for 72 h in vitro	[65]
	Silicone Hydrogel Lenses	UC-MSC-loaded silicone hydrogel lenses	Rabbit model of high-risk corneal transplant rejection	Adaptable to ocular surface curvature; maintains local cell concentration	Ocular retention: Above 4 days; Local cell concentration increased by >10 times	[75]
Combined Treatment (Immunomodulation + Anti-Inflammatory Therapy)	6-Shogaol + Anti-Inflammatory Effects	6-Shogaol + HIF-1α pathway targeting	Endotoxin-induced uveitis (EIU) mice, BV2 cells	Synergistically inhibits endoplasmic reticulum stress and inflammation; targets HIF-1α pathway	No obvious complications; reduces pro-inflammatory cytokine release	[19]
	Lipoxin A4/B4 + CXCR3 Antagonism	Lipoxin A4/B4 + CXCR3 antagonist	LPS-induced uveitis mice	Synergistically inhibits glial cell activation; enhances inflammation resolution	No obvious complications; reduces retinal inflammatory infiltration	[34]
	Artesunate + Metabolic Regulation	Artesunate + metabolic microenvironment modulation	Chronic recurrent EAU (tEAU) rats	Improves Treg metabolic microenvironment; reduces disease recurrence rate	No obvious complications; enhances Treg functional stability	[52]
	IL-2 + Rapamycin	Low-dose IL-2 + Rapamycin	Mouse skin transplantation model (corneal transplantation-related)	Synergistically expands Treg cells; inhibits effector T cell activation	Superior to single-drug therapy in delaying graft rejection; no clear ocular-specific data	[64]
	PKHB1-MN + Antiviral Drugs	PKHB1 peptide-loaded soluble microneedle patch + Ganciclovir	HSV keratitis (HSK) mouse model	Enhances antiviral immunity; reduces drug resistance risk; improves Treg function	Microneedle height: 200 μm; Base diameter: 100 μm; Drug retention: 12 h	[78]

## Data Availability

No new data were created or analyzed in this study.
